# Comparison between external locking plate fixation and conventional external fixation for extraarticular proximal tibial fractures: a finite element analysis

**DOI:** 10.1186/s13018-021-02907-3

**Published:** 2022-01-11

**Authors:** Dejan Blažević, Janoš Kodvanj, Petra Adamović, Dinko Vidović, Zlatko Trobonjača, Srećko Sabalić

**Affiliations:** 1 Department of Traumatology, Sestre milosrdnice University Hospital Center, Draškovićeva 19, 10000 Zagreb, Croatia; 2 University of Applied Health Sciences, Mlinarska cesta 38, 10000 Zagreb, Croatia; 3 Faculty of Mechanical Engineering and Naval Architecture, University of Zagreb, Ivana Lučića 5, 10002 Zagreb, Croatia; 4 School of Dental Medicine, University of Zagreb, Gundulićeva 5, 10000 Zagreb, Croatia; 5 School of Medicine, University of Rijeka, Braće Branchetta 20/1, 51000 Rijeka, Croatia; 6 School of Medicine, University of Split, Šoltanska 2, 21000 Split, Croatia

**Keywords:** Finite element analysis, Locking plate, External fixator, Tibia, Fracture

## Abstract

**Background:**

Good clinical outcomes for locking plates as an external fixator to treat tibial fractures have been reported. However, external locking plate fixation is still generally rarely performed. This study aimed to compare the stability of an external locking plate fixator with that of a conventional external fixator for extraarticular proximal tibial fractures using finite element analysis.

**Methods:**

Three models were constructed: (1) external locking plate fixation of proximal tibial fracture with lateral proximal tibial locking plate and 5-mm screws (ELP), (2) conventional external fixation of proximal tibial fracture with an 11-mm rod and 5-mm Schanz screws (EF-11), and (3) conventional external fixation of a proximal tibial fracture with a 7-mm rod and 5-mm Schanz screws (EF-7). The stress distribution, displacement at the fracture gap, and stiffness of the three finite element models at 30-, 40-, 50-, and 60-mm plate–rod offsets from the lateral surface of the lateral condyle of the tibia were determined.

**Results:**

The conventional external fixator showed higher stiffness than the external locking plate fixator. In all models, the stiffness decreased as the distance of the plate–rod from the bone surface increased. The maximum stiffness was 121.06 N/mm in the EF-11 model with 30-mm tibia–rod offset. In the EF-7 model group, the maximum stiffness was 40.00 N/mm in the model with 30-mm tibia–rod offset. In the ELP model group, the maximum stiffness was 35.79 N/mm in the model with 30-mm tibia–plate offset.

**Conclusions:**

Finite element analysis indicated that external locking plate fixation is more flexible than conventional external fixation and can influence secondary bone healing. External locking plate fixation requires the placement of the plate as close as possible to the skin, which allows for a low-profile design because the increased distance from the plate to the bone can be too flexible for bone healing. Further experimental mechanical model tests are necessary to validate these finite element models, and further biological analysis is necessary to evaluate the effect of external locking plate fixation on fracture healing.

## Background

Proximal tibial fracture, which can be associated with severe soft tissue injuries, requires external fixation [1]. Joint bridging external fixators are usually applied for proximal tibial fractures because it is technically demanding to place a conventional external fixator without bridging the knee. However, most external fixators for the lower extremities are bulky and burdensome for patients [2]. Thus, some clinicians have used locking plates as an external fixator to treat tibial fractures because of their advantages of low profiles and angular stability [3]. The locking plate has axial and angular stability due to the locking-head mechanism and thus forms a unique construction of the plate, screws, and bone [4]. Recent studies have reported good clinical results using external locking plates for treating tibial fractures [5–7]. However, external locking plate fixation is still not generally acknowledged. Furthermore, only a few biomechanical studies of external locking plate fixation have been conducted [8–11]. Thus, this study aimed to compare the stability of an external locking plate fixator with that of a conventional external fixator for extraarticular proximal tibial fractures using finite element analysis.

## Methods

### Three-dimensional modelling

Two-dimensional computed tomography (CT) images were obtained by scanning the composite tibia (Sawbones®, 4th Gen., Composite, 17 PCF Solid Foam Core, Medium) at the Department of Traumatology, Sestre milosrdnice University Hospital Center, Zagreb, Croatia. The CT image slice thicknesses were 0.6 mm in a 512 × 512 matrix. A three-dimensional (3D) model of the tibia was then reconstructed with the CT images using the 3D model reconstruction software Mimics (software version 17.0, Materialise, Leuven, Belgium). The Digital Imaging and Communications in Medicine dataset, which consists of 681 CT images, was imported into Mimics to reconstruct the geometry of the tibia, including the contours of the cortical and cancellous bone.

**Fig. 1:**
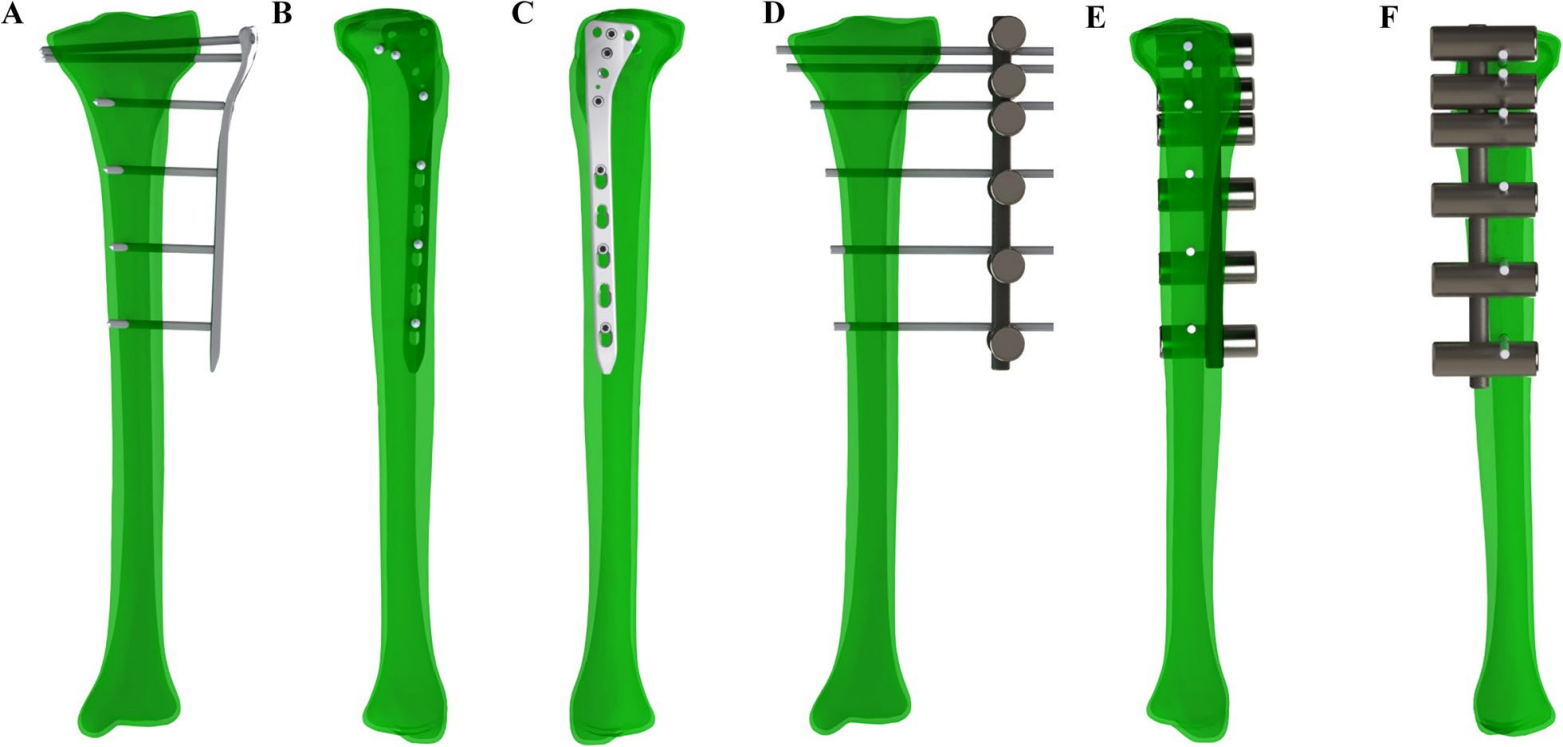
Position of the screws in the ELP model and the EF model: **A** anterior view of the ELP model, **B** medial view of the ELP model, **C** lateral view of the ELP model, **D** anterior view of the EF model, **E** medial view of the EF model, and **F** lateral view of the EF model

The 3D models of the lateral proximal tibial locking plate and screws were designed according to the manufacturer’s specifications (Instrumentaria, Zagreb, Croatia) using computer-aided design (CAD) software (SOLIDWORKS 2017, Dassault Systèmes, Massachusetts, USA). The moment of inertia of the lateral proximal tibial locking plate measured at the cross-sectional area between the locking screw holes and the combi screw holes was 120.28 mm^4^. The 3D models of conventional external fixators with Schanz pins, rods, and clamps were also designed using CAD software. The moments of inertia of the 7-mm rod and the 11-mm rod were 117.86 mm^4^ and 718.69 mm^4^, respectively. Three models were constructed using CAD software: (1) external locking plate fixation of a proximal tibial fracture with a lateral proximal tibial locking plate and 5-mm screws (ELP), (2) conventional external fixation of a proximal tibial fracture with an 11-mm rod and 5-mm Schanz screws (EF-11), and (3) conventional external fixation of a proximal tibial fracture with a 7-mm rod and 5-mm Schanz screws (EF-7). The ELP model was computed with 30-, 40-, 50-, and 60-mm plate offset from the lateral surface of the lateral condyles of the tibia. In addition, the EF-11 and EF-7 models were computed with 30-, 40-, 50-, and 60-mm rod offsets from the lateral surface of the lateral condyle of the tibia. The geometry of the locking screws and the Schanz screws was simplified to a cylinder (*D* = 5 mm) to conserve computing time. Figure 1 shows the position of the three screws in the proximal tibia and three screws in the tibial diaphysis in the ELP model and the EF model. Distances between screws in the ELP model were similar to distances between screws in the EF model, despite the second screw from the proximal. Additionally, the first and second screws of the ELP model were in the more posterior position than the first and second screws of the EF model. In the EF model, all screws were in the linear position. Design of the lateral proximal tibial locking plate determined the deviation of the first two screws from the proximal tibia in the ELP model.

### Finite element modelling

After alignment of the tibia and bone fixation systems in the CAD software, the entire solid models were imported into commercial finite element software (Abaqus/CAE 6.14-5, Dassault Systèmes, Simulia Corp, Rhode Island, USA). In all models, a 10-mm fracture gap was created between 60 and 70 mm beneath the tibial plateau, which represents a multifragmentary extraarticular proximal tibia fracture (AO/OTA classification: 41A3.3) [12]. All models were fixed with three proximal screws and three distal screws from the fracture gap. All materials were assumed to have linear elastic, homogeneous, and isotropic properties. The Young’s modulus of TiAl6V4 was set to 110 GPa, and the Poisson’s ratio was set to 0.3 for the material properties of the locking plate, locking screws, rods, clamps, and Schanz screws [13, 14]. For the material properties of the tibia, we set the Young’s moduli to 17 GPa and 1.1 GPa for the cortical and cancellous bone, respectively [13, 15, 16]. Poisson’s ratio for both bone types was 0.3 [15, 16]. Tied constraints were applied between the locking screws and the bone, the locking screws and the locking plate, the Schanz pins and the bone, and the rod, the clamps and the Schanz pins.

The finite element models were meshed with ten node quadratic tetrahedral elements. The total number of elements in the finite models ranged from 1,134,416 to 1,413,755, and the total number of nodes ranged from 1,703,188 to 2,111,440, depending on the model.

### Boundary conditions

**Fig. 2:**
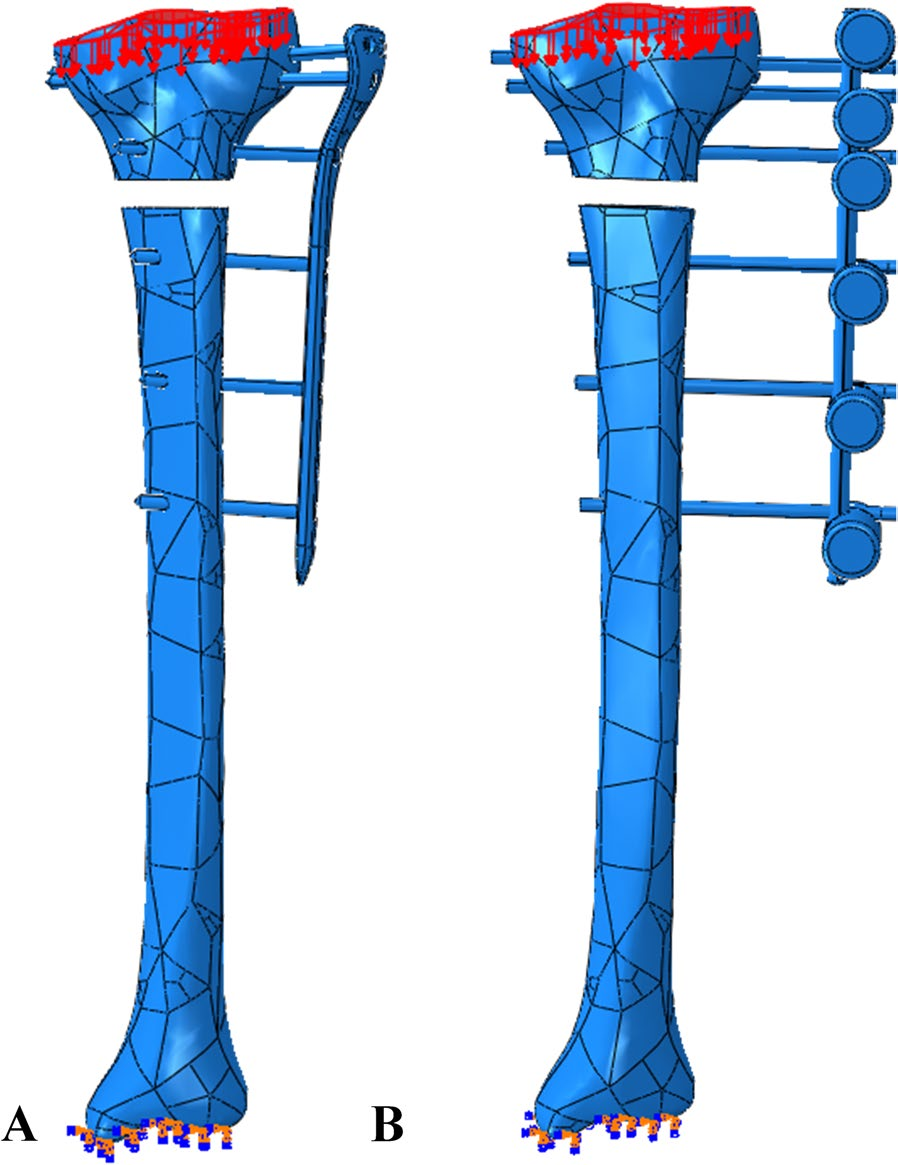
Boundary conditions: **A** external locking plate fixator and **B** conventional external fixator

The finite element model generated outer cortical and inner cancellous bones, indicating its validity; thus, the material properties were assigned accordingly. In all models, an axial load of 50 N was applied to the surface of the tibial plateau in a proximal to distal direction, which represents toe-touch weight bearing [17]. To prevent rigid body motion during the analysis, the tibial plafond was fixed in all degrees of freedom (Fig. 2). The stress distribution, displacement at the fracture gap, and stiffness of the three finite element models with 30-, 40-, 50-, and 60-mm plate–rod offsets from the lateral surface of the lateral condyle of the tibia were obtained.

### Mesh sensitivity analysis

**Fig. 3:**
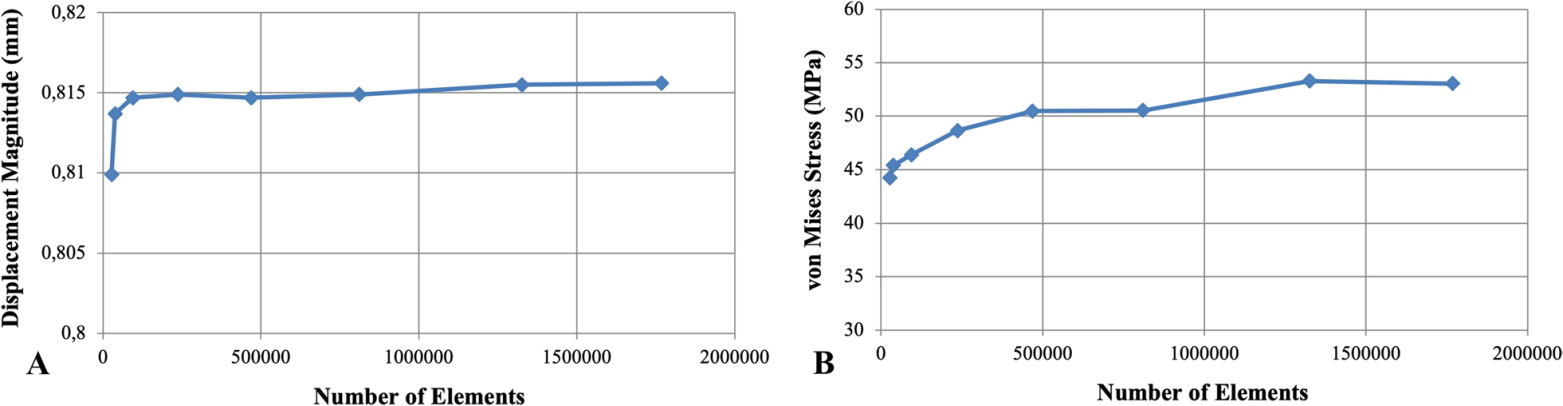
Mesh sensitivity analysis: **A** comparing the displacement magnitude and the number of elements and **B** comparing the Von Mises stress values and the number of elements

A convergence study was performed to establish the appropriate mesh refinement. Mesh convergence analysis was performed by using a model of an intact cortical tibia. The finite element model was optimized and joined through convergence analysis by using the h-refinement method. Both mesh sensitivity analysis between displacement magnitude and number of elements (Fig. 3A) and mesh sensitivity analysis between von Mises stress values and number of elements (Fig. 3B) showed that convergence was achieved.

## Results

### Stress distribution

**Fig. 4:**
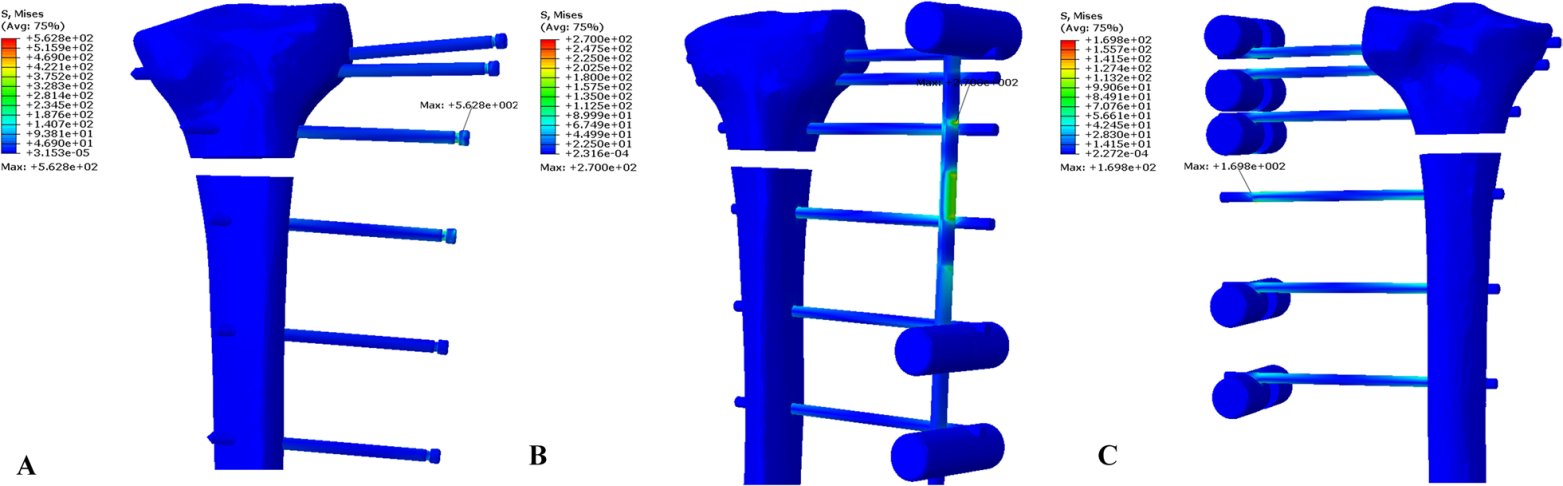
Maximum von Mises stress: **A** the ELP model with a 60 mm tibia–plate offset, **B** the EF-7 model with a 60 mm tibia–rod offset, and **C** the EF-11 model with a 60 mm tibia–rod offset

In the ELP model, the maximum von Mises stress was 562.8 MPa, observed in the nearest screw to the fracture gap on the proximal side of the fracture gap, in the model with a 60-mm tibia–plate offset (Fig. 4A). In the EF-7 model group, the maximum von Mises stress was 270 MPa in the rod, observed around the Schanz screw nearest to the fracture gap on the proximal side of the fracture gap in the model with a 60-mm tibia–rod offset (Fig. 4B). In the EF-11 model group, the maximum von Mises stress was 169.8 MPa, observed in the Schanz screw nearest the fracture gap on the distal side of the fracture gap in the model with a 60-mm tibia–rod offset (Fig. 4C). Table 1 shows the distribution of the maximum von Mises stress in all model groups.

**Table 1:** Distribution of the maximum von Mises stress in the three model groups

Plate–rod offset (mm)	Maximum von Mises stress (MPa)		
	ELP model	EF-7 model	EF-11 model
30	377.9	191.8	138.2
40	444.7	236.2	146.6
50	494.7	241	163.5
60	562.8	270	169.8

### Displacement

**Fig. 5:**
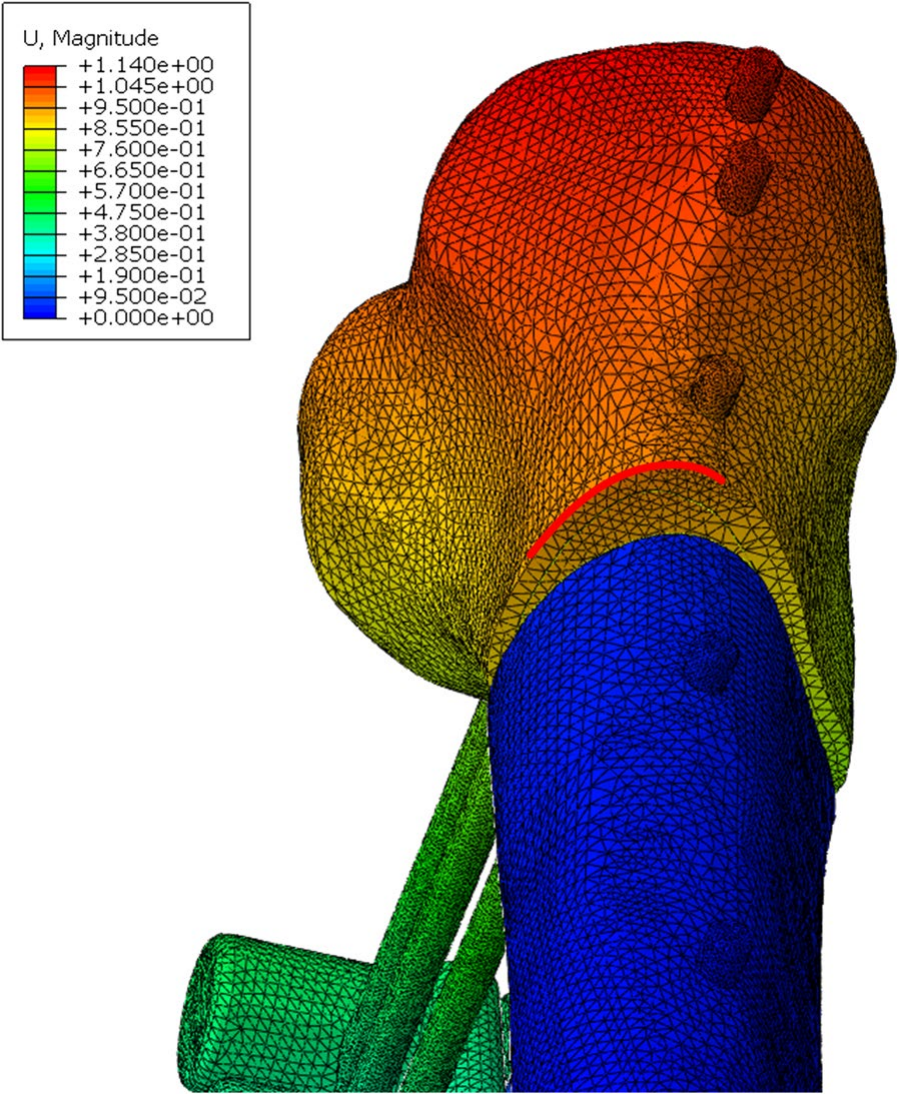
Medial border of the tibia at the proximal site to the fracture gap where the displacement was measured, indicated by the red area

**Fig. 6:**
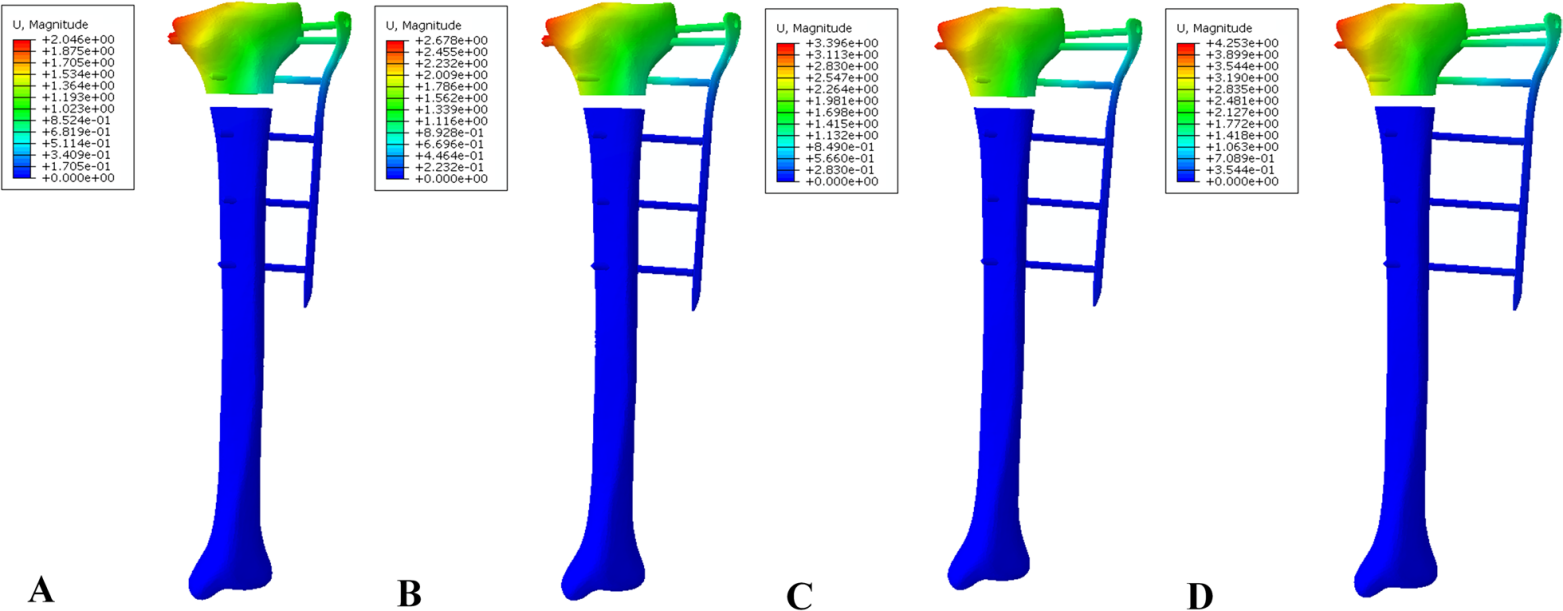
Distribution of displacement in the ELP model group: **A** 30-mm offset, **B** 40-mm offset, **C** 50-mm offset, and **D** 60-mm offset

**Fig. 7:**
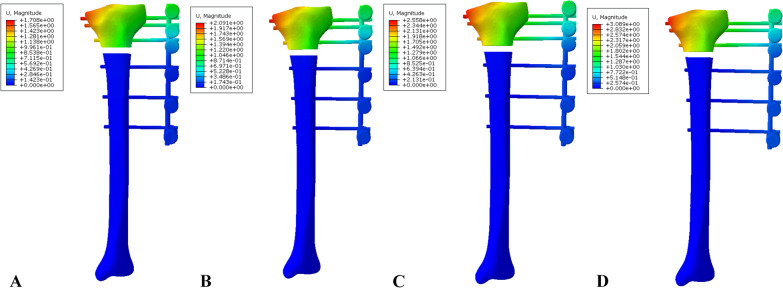
Distribution of displacement in the EF-7 model group: **A** 30-mm offset, **B** 40-mm offset, **C** 50-mm offset, and **D** 60-mm offset

**Fig. 8:**
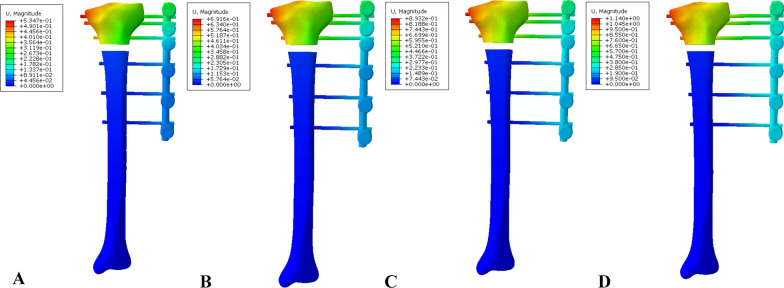
Distribution of displacement in the EF-11 model group: **A** 30-mm offset, **B** 40-mm offset, **C** 50-mm offset, and **D** 60-mm offset

Displacement was measured at the medial border of the tibia at the side proximal to the fracture gap (Fig. 5). The maximum displacement was 3.281 mm in the ELP model with a 60-mm tibia–plate offset. In the EF-7 model group, the maximum displacement was 2.523 mm in the model with a 60-mm tibia–rod offset. In the EF-11 model group, the maximum displacement was 0.984 mm in the model with a 60-mm tibia–rod offset. Table 2 shows the distribution of the displacement in all model groups. Figures 6, 7, and 8 show the displacement distribution for all model groups.

**Table 2:** Distribution of the displacement at the medial border of the tibia at the proximal site to the fracture gap in all model groups

Plate–rod offset (mm)	Displacement (mm)		
	ELP model	EF-7 model	EF-11 model
30	1.397	1.250	0.413
40	1.913	1.610	0.563
50	2.542	2.033	0.751
60	3.281	2.523	0.984

### Stiffness

The maximum stiffness was 121.06 N/mm in the EF-11 model with a 30-mm tibia–rod offset. In the EF-7 model group, the maximum stiffness was 40.00 N/mm in the model with a 30-mm tibia–rod offset. In the ELP model group, the maximum stiffness was 35.79 N/mm in the model with a 30-mm tibia–plate offset. Table 3 shows the distribution of the stiffness in all model groups.

**Table 3:** Distribution of the stiffness in the three model groups

Plate–rod offset (mm)	Stiffness (N/mm)		
	ELP model	EF-7 model	EF-11 model
30	35.79	40.00	121.06
40	26.14	31.05	88.81
50	19.67	24.59	66.58
60	15.24	19.82	50.81

## Discussion

Evidence concerning the biomechanical characteristics of external locking plate fixation is still inadequate to support its clinical recommendations as an external locking plate. Our study showed higher stiffness of the conventional external fixator than that of the external locking plate fixator. In all models, the stiffness decreased as the distance between the plate/rod and the bone surface increased. To the best of our knowledge, this is the first finite element analysis comparing an external locking plate fixator and a conventional external fixator for proximal tibial fractures.

Ideal osteosynthesis involves the optimal balance between biology and mechanics that promotes fracture healing. The concept of biological fracture fixation implies preserving soft tissue and periosteal blood supply and achieving relative stability that promotes callus formation [18]. Internal locking plate fixation can be too stiff to promote optimal fracture healing by callus formation or can cause inconsistent and asymmetric formation of the periosteal callus [19]. Bottlang et al. introduced a modified internal locked plating technology, termed “far cortical locking,” in 2010 [20]. In this technology, elastic fixation is achieved through cantilevered bending of the far cortical locking screw shafts. The mechanism is similar to an external fixator that derives elasticity from fixation pin flexion. Compared with locked plating internal constructs, far cortical locking internal constructs form more calli by providing flexible fixation [20]. External fixators also provide flexible fixation, although fixation that is too flexible can bring instability and nonunion.

Kloen et al. first used the locking compression plate as an external fixator and named this technique “supercutaneous plating” [21]. External locking plate fixators are low-profile external fixators with angular stable screw fixation, facilitating mobilization and providing more comfort and better aesthetics than traditional bar-Schanz pin fixators. Zhang et al. evaluated the outcomes of one-stage external locking plate fixation in 116 tibial fractures [22]. The mean fracture healing times were 12, 20, 14, and 24 weeks for proximal, shaft, distal, and multisegmental tibial fractures, respectively [22]. Luo et al. conducted a systematic review of 12 studies and reported that external locking plate fixation achieved satisfactory functional outcomes and union rates and low complication rates [3].

However, the few biomechanical studies that investigated the biomechanical aspects of external locking plate fixation were heterogeneous [8–11]. Zhang et al. reported a finite element analysis of external locking plate fixation with a contralateral femoral less invasive stabilization system (LISS) and different plate–bone distances (1, 10, 20, and 30 mm) in distal tibial metaphyseal fractures [23]. They concluded that the construct with a 30 mm plate–bone distance might be beneficial to induce callus formation. Furthermore, more profound increases in stiffness were observed in the 1-, 10-, and 20-mm groups, indicating the potential of load shielding [23]. Ma et al. conducted a finite element analysis to evaluate the biomechanical performance of external and internal locking plate fixation of proximal tibial fractures with a LISS plate [24]. They showed that compared to the internal locking plate model, the axial stiffness was reduced by 84% for the external locking plate model with a 6-cm offset and by 94% for the external locking plate model with a 10-cm offset [24]. In the clinical application of external fixation, the distance of the external fixator from the bone depends on soft tissue swelling and the individual soft tissue thickness. Therefore, finite element modelling was performed in this study with 30-, 40-, 50- and 60-mm plate/rod offsets from the lateral surface of the lateral condyle of the tibia due to the skin and soft-tissue thickness individuality.

In our study, increasing the distance of the plate or rod from the bone surface from 30 to 60 mm uniformly reduced the stiffness by more than 50% in all models. The stiffness of the ELP model with a 30-mm tibia plate offset was 57.42% higher than that of the ELP model with a 60-mm tibia plate offset. The stiffness of the EF-7 model with a 30-mm tibia plate offset was 50.45% higher than that of the EF-7 model with a 60-mm tibia plate offset. The stiffness of the EF-11 model with a 30-mm tibia plate offset was 58.03% higher than that of the EF-11 model with a 60-mm tibia plate offset.

Ang et al. showed no statistically significant differences between the torsional stiffness of the external titanium locking plate (0.639 Nm/degree) and the unilateral external fixator (0.512 Nm/degree) in the composite tibia model of comminuted mid-shaft tibia fractures with 20-mm plate or rod-bone distances [9]. Zhang et al. evaluated a model of tibial distal metaphyseal fractures externally fixed with a femoral LISS plate under axial load (simulating standing with full weight bearing), axial load and internal torsion force (simulating standing with full weight bearing accompanied by the trunk rotating towards the opposite side), and axial load and external torsion force (simulating standing with full weight bearing accompanied by the trunk rotating towards the ipsilateral side) [23]. They concluded that patients should be warned to avoid rotation of the lower leg if permitted to bear full weight immediately after the procedure [23]. In our study, an isolated axial load of 50 N was applied to the surface of the tibial plateau in the direction from proximal to distal. Our loading settings represent toe-touch weight bearing, which is mainly axial load force without torsional force.

Kanchanomai et al. evaluated the effects of fracture gap sizes (1, 5, and 10 mm) on the stability of external locking plate fixation with 30-mm plate-bone distances in a composite tibial shaft fracture model [11]. The stiffness of the 10-mm fracture gap model, which represents a 10-mm comminuted fracture on the midshaft of the tibia, was significantly lower than that of the 5-mm fracture gap model [11]. The stiffness of the 5-mm fracture gap model was significantly lower than that of the 1-mm fracture gap model, which represents a transverse fracture on the midshaft of the tibia [11]. In our study, a 10-mm fracture gap represented a multifragmentary extraarticular proximal tibia fracture (AO/OTA classification: 41A3.3) [12].

Liu et al. compared the axial stiffness of distal tibial internal locking plates (177.9 ± 20.31 N/mm), distal tibial external locking plates with a 30-mm plate-bone distance (25.04 ± 2.19 N/mm), and distal femur external locking plates with a 30-mm plate-bone distance (84.38 ± 14.37 N/mm) in distal tibial fracture composite models in biomechanics tests [8]. The distal femur locking plate is thicker than the distal tibial locking plate; thus, distal femur external locking plate fixation is stiffer than distal tibial external locking plate fixation. In our finite element analysis, the axial stiffness of the proximal tibial external locking plate with a 30-mm plate-bone distance was 35.79 N/mm in the proximal tibial fracture model, which is comparable with the axial stiffness of distal tibial external locking plates with 30-mm plate-bone distances (25.04 ± 2.19 N/mm) in the distal tibial fracture composite model [8].

In our study, the contact body between the locking screws and the bone, the locking screws and the locking plate, the Schanz pins and the bone, and the rod, clamps and the Schanz pins were set as tied constraints. With regard to tied constraints, the stiffness of the models was most affected by the moment of inertia of the plate or rod, which was 120.28 mm^4^ for the plate. The moments of inertia of the 7-mm rod and the 11-mm rod were 117.86 mm^4^ and 718.69 mm^4^, respectively. The moment of inertia of the 11-mm rod was 83.26% higher than that of the plate. The stiffness of the EF-11 model with a 30-mm tibia–rod offset was 70.44% higher than that of the ELP model with a 30-mm tibia–plate offset. The stiffness of the EF-7 model with a 30-mm tibia–rod offset was 10.52% higher than that of the ELP model with a 30-mm tibia–plate offset. The ELP model was more flexible than the EF-11 model due to its lower moment of inertia. Furthermore, the stiffness of the ELP model can be improved by increasing the thickness of the lateral proximal tibial locking plate, which in turn leads to an increase in the moment of inertia of the plate.

Ma et al. compared the axial stiffness of distal femur internal locking plates (347.06 ± 17.06 N/mm), distal femur external locking plates with 60-mm plate-bone distances (66.75 ± 7.95), and conventional external fixators (22.80 ± 2.10 N/mm) in proximal tibial fracture composite models using biomechanics tests [10]. The conventional external fixator was applied in a triangle configuration with two rods 60 mm from the bone, two Schanz screws inserted bilaterally in the proximal tibia and three collinear Schanz screws inserted in the distal tibia [10]. A distal femur external locking plate was applied with four screws in the proximal tibia and three screws in the diaphysis region [10]. Therefore, in the biomechanical study by Ma et al. [10], the configuration of distal femur external locking plate fixation of the proximal tibial fracture was biomechanically more favourable than conventional external fixator with distal Schanz screws inserted far from the fracture gap. In our finite element analysis, the conventional external fixator was modelled with three Schanz screws in the proximal tibia and three Schanz screws in the diaphysis region, similar to the screw position of external locking plate fixation. Thus, credible models of conventional external fixation and external locking plate fixation for comparison were obtained in our study.

The distance of the screws from the fracture gap, the distance between the screws, the thickness of the rod or plate, the diameter of the screws, and the distance of the rod or plate from the bone are factors that affect the stability of the external fixation constructs.

The limitations of the present study must be considered. One limitation is that contact interfaces were tied constraints between the different fixator components and the bone. Karunratanakul et al. showed that contact settings between the different fixator components are highly predictive of the external fixator stiffness [25]. We compared the external locking plate fixator with the conventional external fixator under ideal contact settings because it is difficult to determine the real locking screw-plate and clamp-rod-Schanz screw contact settings without an experimental validation study. However, with the limitation that no experimental validation was conducted, a comparison between external locking plate fixators and conventional external fixators for proximal tibial fractures requires more research. Furthermore, the results of the present study can be considered evidence to compare external locking plate fixators and conventional external fixators in mechanical models for the validity of their biomechanical properties and may facilitate further mechanical research. The second limitation is that two-dimensional CT images were obtained by scanning the composite tibia despite living bone. However, the use of a commercialized composite model (i.e. Sawbones) appears to be an acceptable practice to validate finite element models [26]. Fourth-generation composite bones have average stiffnesses and strains that are in the range for natural bones [15]. The third limitation of this study is that finite element analysis cannot evaluate the dynamic stability of models, which is important for understanding the effect of the fixator on fracture healing. Manipulation of the mechanical environment is important to optimize and accelerate fracture healing. One concept is reverse dynamization, which postulates that the fracture should initially be stabilized with flexible fixation to promote cartilaginous callus formation [27]. This should be followed by more rigid fixation after adequate fracture callus formation to accelerate healing and the remodelling process [27]. Further experimental fatigue tests with external locking plate fixator–composite tibia models and conventional external fixator–composite tibia models should be performed to determine the influence of the locking screw–and clamp–rod–Schanz screw contact settings on the dynamic stability of each model.

## Conclusions

Finite element analysis indicated that external locking plate fixation is more flexible than conventional external fixation and can influence secondary bone healing. External locking plate fixation requires the placement of the plate as close as possible to the skin, which allows low-profile design because the increased distance of the plate from bone can be too flexible for bone healing. Further experimental mechanical model tests are necessary to validate these finite element models, and further biological analysis is necessary to evaluate the effect of external locking plate fixation on fracture healing.

## Data Availability

The datasets used and/or analysed during the current study are available from the corresponding author on reasonable request.

## Abbreviations

3D: Three-dimensional
CAD: Computer-aided design
CT: Computed tomography
ELP: External locking plating of proximal tibial fracture with lateral proximal tibial locking plate and 5-mm screws
EF-11: Conventional external fixation of proximal tibial fracture with an 11-mm rod and 5-mm Schanz screws
EF-7: Conventional external fixation of proximal tibial fracture with a 7-mm rod and 5-mm Schanz screws
LISS: Less invasive stabilization system
